# Diagnostic excellence in primary care

**DOI:** 10.1002/jgf2.617

**Published:** 2023-03-20

**Authors:** Takashi Watari, Gordon D. Schiff

**Affiliations:** ^1^ General Medicine Center Shimane University Hospital Shimane Japan; ^2^ Department of Medicine University of Michigan Medical School Ann Arbor Michigan USA; ^3^ Center for Primary Care Harvard Medical School Boston Massachusetts USA; ^4^ Center for Patient Safety Research Brigham and Women's Hospital Boston Massachusetts USA

## Abstract

Diagnostic excellence is based on six fundamental principles of healthcare quality proposed by the Institute of Medicine in 2001, which state that diagnoses must be safe, effective, patient‐centered, timely, efficient, and equitable.

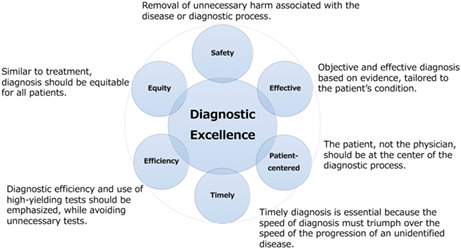

The intellectual task of diagnosis performed by medical professionals is a critical decision‐making process that is vital for determining a patient's treatment and prognosis. Therefore, diagnostics play a pivotal role in healthcare, particularly in primary care, where patients often present with undifferentiated symptoms that the clinician sorts out and accurately diagnoses. According to *William Osler*, “medicine is a science of uncertainty and the art of probability.” This highlights the creative artistic aspect of medicine, as well as the fact that diagnosis is not always certain or even correct.

Since the publication of “To err is human” in 1999, and more recently in 2015 improving diagnosis in healthcare, the potential for error and need to improve diagnostic safety has been increasingly recognized.^1^ Currently, viewing diagnosis through a patient safety lens and striving for diagnostic excellence has significantly changed the field.^2^, ^3^ Diagnostic excellence is based on six fundamental principles of healthcare quality proposed by the Institute of Medicine in 2001, which state that diagnoses must be safe, effective, patient‐centered, timely, efficient, and equitable (Figure 1).^2^ In summary, it refers to an optimal diagnostic process that accurately describes a patient's condition.^2^ Internal medicine has always focused on the evaluation of patients presenting with symptoms and signs and the informed and appropriate ordering of diagnostic tests. This includes their cost‐effectiveness, with the goal of providing an explanation of the patients' history, physical examination, and laboratory, radiology, and other tests.^3^ Although an ancient art, the application of safety science is a relatively new concept for achieving diagnostic excellence and is well suited as a research topic for general medicine physicians working in primary care.

**FIGURE 1:**
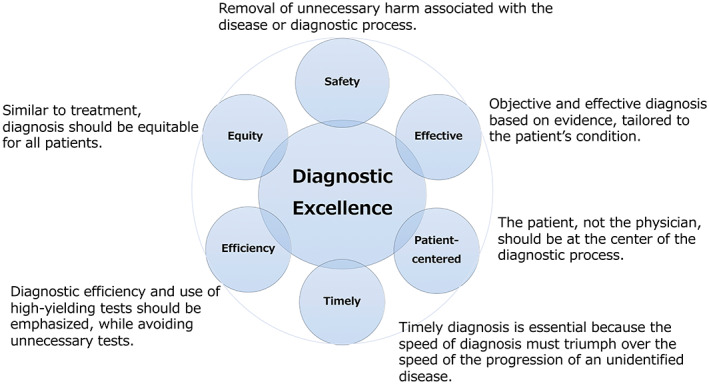
Six dimensions of healthcare quality embraced by diagnostic excellence as enumerated by the Institute of Medicine in 2001.

First, diagnostic excellence has the foundational principles of patient‐centeredness and patient engagement, which are important pillars of primary healthcare. A truly “patient‐centered diagnosis” must be based on excellent communication that respects the patient's needs, values, opinions about the disease, and life context. This is precisely the principle of patient‐centeredness and continuity of care, which is highly prioritized by primary care. Furthermore, only continuous dialogue, follow‐up conversations, and monitoring between physicians and patients can address diagnostic uncertainty. To be effective, patients must be engaged in coproducing the diagnosis by presenting them to their clinicians in a timely manner, accurately describing their symptoms, recounting their time course, engaging in shared decision‐making regarding diagnostic possibilities and follow‐up, and adhering to monitoring and follow‐up plans.

Second, the synergy between the four core functions (4Cs) of primary care and the comprehensiveness inherent in diagnostic excellence is exceptionally high. The 4Cs of primary care essential for quality healthcare include first contact, comprehensiveness, coordination, and continuity. Its achievement not only improves health outcomes for patients but also improves inequalities and cost‐effectiveness.^4^ This definition is consistent with the foundation of diagnostic excellence.^2^ To date, research on the diagnostic process has focused on the cycle of collecting and interpreting information for accurate diagnosis and recalling differential diagnoses. However, the diagnostic journey of a patient encompasses many elements that exist before the patient experiences a health problem, along with the patient's family relationships and social context.^5^ Therefore, we need to understand that new diagnostic paradigms in diagnostic research must move away from a discipline that mainly aims to list differential diagnoses and improve the accuracy of diagnosis, to one that is more patient‐centered and has a social sciences aspect.

Furthermore, diagnostic excellence encompasses diagnostic equity.^5^ McDonald states that inequalities in the diagnostic process lead to inequalities in medical outcomes.^5^ Specifically, various group attributes and characteristics, including age, sex, race, poverty and handicaps, (e.g., belonging to a sexual minority, or living with a physical disability) can increase the risk of diagnostic inequity, leading to inequitable health outcomes.^5^ For a deeper understanding, physicians need to look beyond the superficial level of listing diagnoses to better understand the underlying etiologies, both clinically and environmentally. This includes a better understanding of the social determinants of diagnosis and disease (SDoH). SDoH is a nonmedical factor that influences health outcomes. It has been estimated that socioeconomic and behavioral factors determine more than half of health outcomes. Physicians should be aware of the socioeconomic context of patients and their surroundings and should play a role in providing quality patient‐centered diagnosis and care to address health inequities. Likewise, they need to understand the contextual and environmental factors that define their diagnostic work. Primary care physicians' mindsets are highly compatible with the inclusion of diagnostic excellence.

Donald M. Berwick states that “diagnostic excellence means that the needs of the patient, for solace and relief, come first.”^3^ The essential purpose of a diagnosis is to accurately classify the cause of a patient's impending distress based on the latest understanding of biology and psychology. Thus, diagnostic excellence is a complicated process that integrates the scientific attributional concepts of clinical medicine with a patient's culture, worldview, and knowledge. Additionally, it integrates the biopsychosocial model of care, which is the most important family medicine approach routinely used by primary care physicians.^3^ As primary care physicians, we must work to achieve diagnostic excellence while preventing errors through patient‐centered diagnostic processes.

## AUTHOR CONTRIBUTIONS

All authors have access to the information utilized and participated in the preparation of this manuscript.

## CONFLICT OF INTEREST STATEMENT

None.

## CONSENT FOR PUBLICATION

All authors have provided their consent for publication.

## Data Availability

All relevant data are included in this report.

## References

[1] Watari T , Tokuda Y . Role of Japan's general physicians in healthcare quality improvement and patient safety. J Gen Fam Med. 2022;23(3):137–9. 10.1002/jgf2.541 35509337 PMC9062560

[2] Yang D , Fineberg HV , Cosby K . Diagnostic excellence. JAMA. 2021;326(19):1905–6. 10.1001/jama.2021.19493 34709367

[3] Berwick DM . Diagnostic excellence through the lens of patient‐centeredness. JAMA. 2021;326(21):2127–8. 10.1001/jama.2021.19513 34792525

[4] Jimenez G , Matchar D , Koh GC , Tyagi S , van der Kleij RM , Chavannes NH , et al. Revisiting the four core functions (4Cs) of primary care: operational definitions and complexities. Prim Health Care Res Dev. 2021;10(22):e68. 10.1017/s1463423621000669 PMC8581591 34753531

[5] McDonald KM . Achieving equity in diagnostic excellence. JAMA. 2022;327(20):1955–6. 10.1001/jama.2022.7252 35522307

